# Resolving the Flap over Bird Wrists

**DOI:** 10.1371/journal.pbio.1001958

**Published:** 2014-09-30

**Authors:** Robin Meadows

**Affiliations:** Freelance Science Writer, Fairfield, California, United States of America

## Abstract

New Developmental Evidence Clarifies the Evolution of Wrist Bones in the Dinosaur-Bird Transition

Somewhere along the way from early dinosaurs to birds, wrists changed so much that we could be excused for thinking birds don't even have them. Wrists went from straight to bent and hyperflexible, allowing birds to fold their wings neatly against their bodies when not flying. Underlying this change is a drop in the number of wrist bones from nine to just four. Paleontology and embryology tell different stories about how this happened, however. Now, in this issue of *PLOS Biology*, Alexander Vargas and colleagues resolve this flap, drawing on both fields to clarify the identity and evolution of bird wrist bones.

The four bones in modern bird wrists are essentially laid out on a two-by-two grid (see Figure 1); if the wing is held up then there are two wrist bones on top next to the hand bones and two on the bottom next to the arm bones. The researchers looked at each of these four bones, starting with the one on the bottom nearest the body. Paleontologists generally call this first bone the radiale, ornithologists call it the scapholunare, and developmental studies variously suggest that it is a single bone (the radiale) or a composite of the radiale and another wrist bone in non-flying dinosaurs called the intermedium.

**Figure 1:**
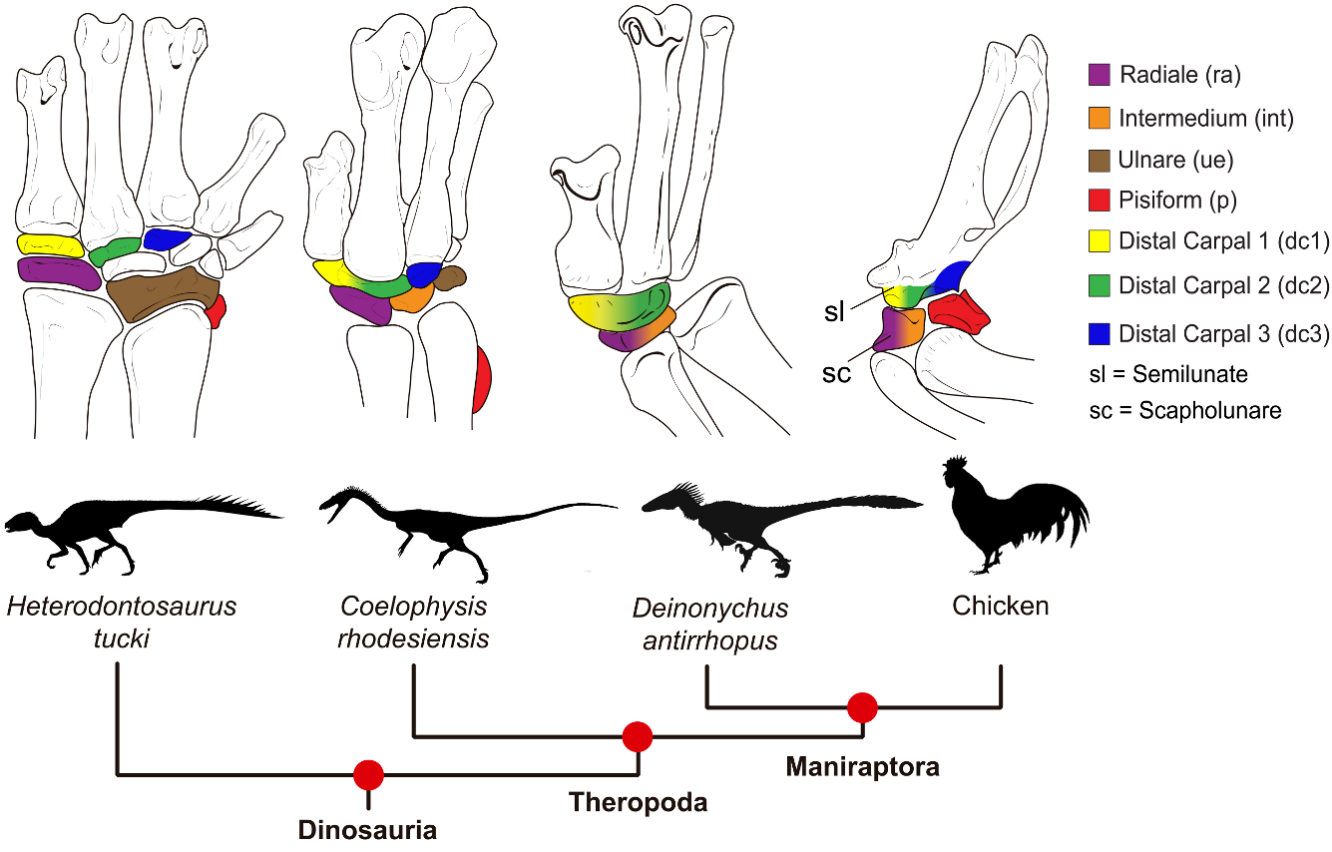
Since early dinosaurs, birds have reduced the number of bones in their wrist, but the origins and identity of those remaining are hard to trace. A new combined study of both embryos and fossils provides a more integrated view of their evolution and a proposed revised nomenclature.

After finding that conventional staining failed to shed light on the identity of the first wrist bone in chicken embryos, the researchers turned to the new technique of whole-mount immunofluorescence. This revealed two distinct regions in the first bone: it has two spots of collagen II expression—which marks cartilage formation—in early embryos, and two spots of collagen IX expression—which marks cartilage maturation—in older embryos.

Importantly, these two regions were also apparent in conventionally stained early embryos of several other bird species, including duck, pigeon, Chilean tinamou, and zebra finch. Taken together, these findings confirm that the radiale and the intermedium fused to form the first bird wrist bone, leading the researchers to recommend that paleontologists join ornithologists in calling it the scapholunare.

The researchers then turned their attention to the bird wrist bone that is on top and nearest the body. Paleontologists call this second bone the semilunate, which in flying dinosaurs is a composite of two non-flying dinosaur wrist bones. In contrast, developmental studies suggest that in birds this second bone is a singleton—called “distal carpal 2”—and this conclusion has cast doubt on the evolution of birds from dinosaurs.

This time, the researchers found that immunofluorescent staining for collagen supported paleontologists, strengthening the link between dinosaurs and birds. Like the first bird wrist bone, the second has two distinct regions of expression of both types of collagen. Accordingly, the researchers suggest that embryologists join paleontologists in calling the second bird wrist bone the semilunate.

Next up is a bone of even greater contention. Some paleontologists call the third bird wrist bone—which is on top and away from the body—“distal carpal 3.” But embryologists believe the third bone is entirely new. They call it “element x” and view it as taking the place of a bone called the ulnare that is lost in bird development.

It is true that the ulnare is fleeting in embryonic birds and is gone completely in adults. But the researchers found that the ulnare and “element x” coexist briefly in embryos of seven bird species, and that the embryonic position of the latter corresponds with that of “distal carpal 3.” These findings refute the notion that “element x” is a new bone that replaces the ulnare. Rather, the researchers suggest that embryologists join paleontologists once again and use the term “distal carpal 3” for the third bird wrist bone.

However, paleontologists didn't win this round completely; they believe bird-like dinosaurs still had the bone that disappears in birds. But the researchers' re-examination of the fossil evidence showed that the ulnare had actually already been lost in the most bird-like dinosaurs, further strengthening the dinosaur–bird link.

Last, the researchers sorted out the fourth and trickiest of the bird wrist bones, which is on the bottom and away from the body. This is the bone that gives the bird wrist its bent shape and extreme outward flexibility. Paleontologists call the fourth bone the ulnare. On the other hand, developmental studies suggest that this bone is the pisiform, which is the wrist version of the kneecap. The fourth bird wrist bone is key to flight, transmitting force on the downstroke while restricting flexibility on the upstroke.

This time, embryologists won. The pisiform is connected by a tendon to a particular muscle in animals from lizards to mammals, and immunofluorescence for the tendon protein tenascin revealed that the same is true in bird embryos.

Paleontologists can be somewhat forgiven for their mistake, however, because the pisiform is not evident in fossils of the most bird-like dinosaurs. The researchers square the paleontological and developmental evidence by proposing that the pisiform was either tiny or failed to ossify in bird-like dinosaurs, but was then re-acquired in birds. Such evolutionary reversals are rare but not unprecedented.

Besides illuminating how birds got the wrists that help them fly, this work shows the benefits of combining paleontology and embryology to piece together evolution. Furthermore, the uneven performance of these fields on their own underscores the downsides of not integrating them. Separately, they can lead down a wrong path—but together, they can come much closer to telling the whole story of evolution.

**Botelho JF, Ossa-Fuentes L, Soto-Acuña S, Smith-Paredes D, Nuñez-León D, et al. (2014) New Developmental Evidence Clarifies the Evolution of Wrist Bones in the Dinosaur–Bird Transition.** doi:10.1371/journal.pbio.1001957

